# Effects of Chinese medicine on patients with acute exacerbations of COPD: study protocol for a randomized controlled trial

**DOI:** 10.1186/s13063-019-3772-y

**Published:** 2019-12-16

**Authors:** Hailong Zhang, Jiansheng Li, Xueqing Yu, Suyun Li, Haifeng Wang, Huanrong Ruan, Yimei Si, Yang Xie, Minghang Wang

**Affiliations:** 1 Department of Respiratory Diseases, The First Affiliated Hospital of Henan University of Chinese Medicine, Zhengzhou, 450000 Henan China; 2 Co-construction Collaborative Innovation Center for Chinese Medicine and Respiratory Diseases by Henan & Education Ministry of P.R. China, Henan University of Chinese Medicine, Zhengzhou, 450046 Henan China; 3 Henan Key Laboratory of Chinese Medicine for Respiratory Disease, Henan University of Chinese Medicine, Zhengzhou, 450046 Henan China

**Keywords:** COPD, Acute exacerbations, Chinese medicine, Randomized controlled trial

## Abstract

**Background:**

The incidence, mortality, and prevalence of chronic obstructive pulmonary disease (COPD) are high in China. Acute exacerbations of COPD (AECOPD) are important events in the management of COPD because they negatively impact health status, rates of hospitalization and readmission, and disease progression. AECOPD have been effectively treated with Chinese medicine for a long time. The aim of this proposed trial is to assess the therapeutic effect of Chinese medicine (CM) on AECOPD.

**Methods/design:**

This proposed study is a multicenter, double-blind, parallel-group randomized controlled trial (RCT). We will randomly assign 378 participants with AECOPD into two groups in a 1:1 ratio. On the basis of health education and conventional treatment, the intervention group will be treated with CM, and the control group is given CM placebo according to CM syndrome. Patients are randomized to either receive CM or placebo, 10 g/packet, twice daily. The double-blind treatment lasts for 2 weeks and is followed up for 4 weeks. The main outcome is the COPD Assessment Test; secondary outcomes are treatment failure rate, treatment success rate, length of hospital stay, AECOPD readmission rate, intubation rate, mortality, dyspnea, the 36-item Short Form Health Survey, and the COPD patient-reported outcome scale. We will document these outcomes faithfully at the beginning of the study, 2 weeks after treatment, and at the 4 weeks follow-up.

**Discussion:**

This high-quality RCT with strict methodology and few design deficits will help to prove the effectiveness of CM for AECOPD. We hope this trial will provide useful evidence for developing a therapeutic schedule with CM for patients with AECOPD.

**Trial registration:**

ClinicalTrials.gov, NCT03428412. Registered on 4 February 2018.

## Background

Chronic obstructive pulmonary disease (COPD) is a common and frequently occurring disease that is greatly harmful to human health. It is currently the fourth leading cause of death in the world [1] but is projected to be the third leading cause of death by 2020. More than 3 million people died of COPD in 2012, accounting for 6% of all deaths globally [2].

Acute exacerbations of COPD (AECOPD) are important events in the management of COPD because they negatively impact health status, rates of hospitalization and readmission, and disease progression. Patients with COPD are susceptible to about 0.5 to 3.5 exacerbations per year. AECOPD is an important factor in the death of COPD patients. In 2013, the total number of COPD deaths in China was about 910,000 and in terms of the number of single diseases ranked No. 3; deaths from COPD accounted for 11% of all deaths in China. Note in particular that the total population with COPD in China accounts for 31.1% of the total number of COPD deaths in the world [3]. COPD is projected to be the fifth economic burden in the world by 2020 [2]. We also point out that AECOPD accounts for a major part of the medical expenses for patients with COPD. The in-hospital mortality for AECOPD was 4.3% with mean costs up to $9545 in the USA in 2006 [4]. The mean cost per admission of hospitalization in patients with AECOPD was up to CNY ¥ 11,598 in China [5]. AECOPD is an important clinical event in COPD and a major determinant of the health status and prognosis of patients with COPD. The early prevention, early detection, scientific understanding, and standardized treatment of AECOPD constitute a major and arduous medical task in clinical practice.

There is a good clinical efficacy of Chinese medicine (CM) for AECOPD patients. However, the experimental design for most clinical studies is unfeasible, the quality of randomized controlled trials (RCTs) is low, and efficacy evaluation indicators are complex and unstandardized [6]. High-quality clinical trials including a scientific design and showing reasonable outcomes on the treatment of AECOPD by CM are urgently needed. Therefore, we conducted a prospective RCT to investigate the effectiveness and safety of CM compared to conventional drugs on patients with AECOPD.

## Methods/design

### Objectives

The study objectives are as follows:

1. Clinical outcome: to test the clinical efficacy of CM for patients with AECOPD
2. Safety evaluation: to evaluate the safety of CM on AECOPD patients.

### Study design

This double-blind, multicenter, parallel-group, superiority, 1:1 randomization ratio prospective clinical study was approved by the Institutional Review Board of the First Affiliated Hospital of Henan University of Chinese Medicine (reference number 2017HL-069-01, validated on 22 November 2017) and registered at ClinicalTrials.gov (NCT03428412). The protocol follows the recommendations of the Standard Protocol Items: Recommendations for Interventional Trials (SPIRIT) initiative (Additional file 1) [7].

**Fig. 1:**
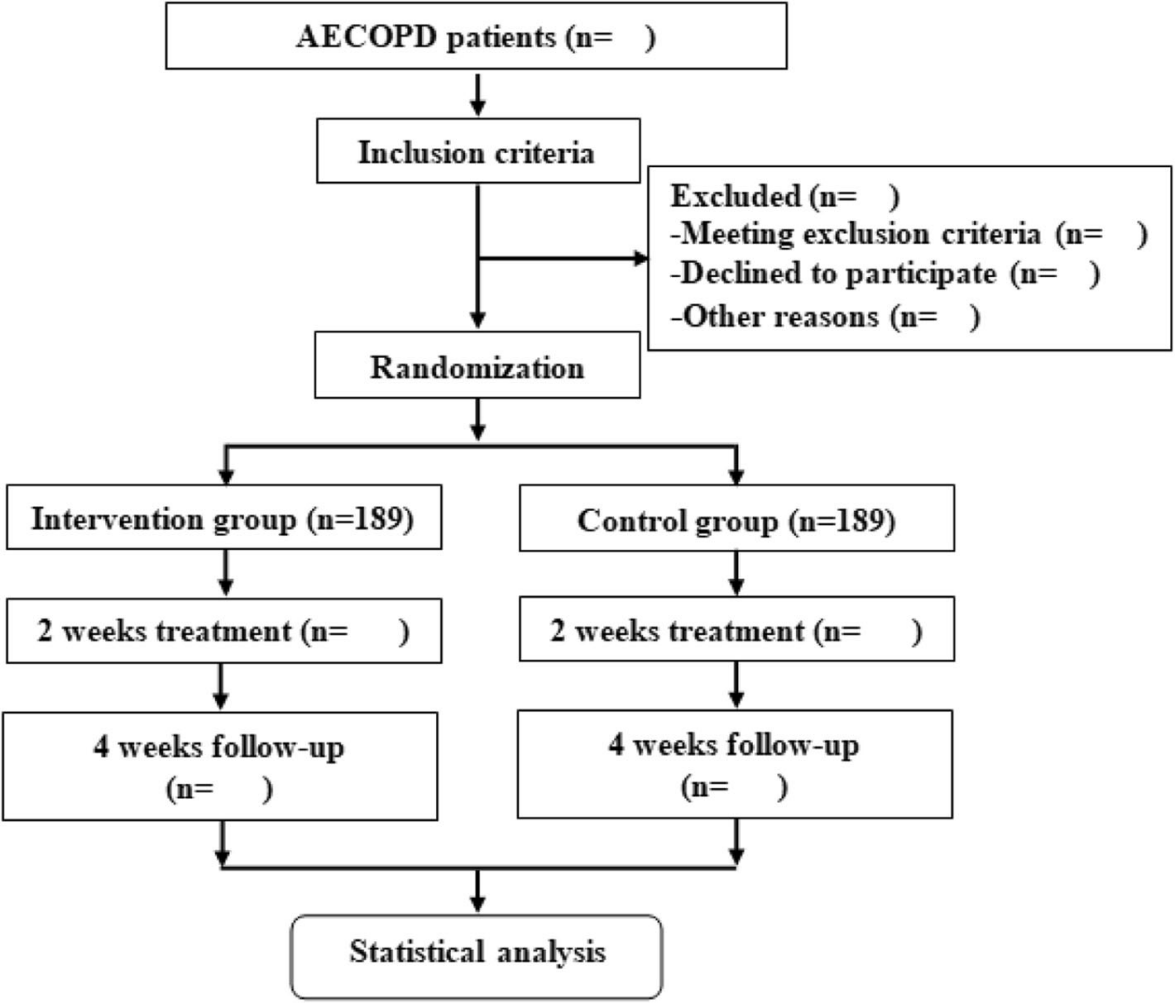
Flowchart for the intervention trial

The flowchart of the study process is detailed in Fig. 1. The timing of treatment visits and data collection is detailed in Table 1.

**Table 1:** Timing of treatment visits and data collection

	Baseline	Treatment phase				Follow-up phase	
	Day 0	Day 4	Day 7	Day 10	Day 14	Day 28	Day 42
	V1	V2	V3	V4	V5	V6	V7
Sign informed consent	×						
Randomization	×						
Medical history	×						
Intervention	×	×	×	×			
Primary outcome							
CAT	×				×	×	×
Secondary outcomes							
Treatment failure		×	×	×	×		
Length of hospital stays					×		
Readmission due to AECOPD						×	×
Intubation rate		×	×	×	×	×	×
Mortality		×	×	×	×	×	×
mMRC	×	×	×	×	×	×	×
SF-36	×				×	×	×
COPD-PRO	×				×	×	×

### Research centers

Patients with AECOPD will be recruited and enrolled from eight research centers in China. They are listed as follows:

- First Affiliated Hospital of Henan University of Chinese Medicine
- Jiangsu Province Hospital of Traditional Chinese Medicine
- First Affiliated Hospital of Zhengzhou University
- Second Affiliated Hospital of Tianjin University of Traditional Chinese Medicine
- Haici Hospital of Qingdao
- People’s Hospital of Zhengzhou
- Shanxi Hospital of Integrated Traditional and Western Medicine
- Nanyang City Center Hospital

### Sample size

The COPD Assessment Test (CAT) will be used as the main outcome. According to previous study results, the average CAT score of AECOPD patients treated by CM is 16.54 ± 5.50 and that of the placebo is 18.33 ± 5.87 [8]. The most reliable estimate of the minimum important difference of the CAT is 2 points [9]. Assuming that α is 0.05 and power is 90%, the two-sided *Z*_1-α/2_ is 1.96 and *Z*_1-_β is 1.28. The standard deviation of the first group (σ_1_) is 5.50 and that of the second group (σ_2_) is 5.78, and the clinically meaningful difference (δ) is 2. Based on the formula $n=\frac{{\left({{Z_{1-}}_{\alpha}}_{/2}+{Z_{1-}}_{\beta}\right)}^2\times \left({\sigma_1}^2+{\sigma_2}^2\right)}{\delta^2}$, the sample size in each group is 170. Considering a 10% dropout rate over the course of the study, 189 patients will be enrolled in each group. The total sample size will be 378.

### Randomization and blinding

A central dynamic stochastic distribution method is adopted. The randomization sequence will be generated by the ‘proc plan’ procedure of SAS software (version 23.0) according to stratified block randomization with a block size of 6, and the random number and group assignment will be obtained from a central randomization system provided by Jiangsu Famous Medical Technology Co., Ltd. in Nanjing, China. Eligible AECOPD patients will be randomly divided into an experimental group and a control group in a 1:1 allocation ratio. The data management and statistical analysis units formulate a random distribution scheme according to the test scheme. They then implement and manage the random distribution scheme through a central random distribution interactive voice operating system. The researcher obtains the subject assignment code via the Internet or phone.

All participants, trial participants, care providers, and outcome assessors except for the data analysts are blind to treatment allocation. The CM placebo packaging in the control group is consistent with the CM granules, and the odor and color are also similar to those of the experimental group CM granules, but there is no clinical effect. If serious adverse events that threaten the safety of participants occur, we will immediately stop the intervention, cancel the blinding, and contact the Institutional Review Board.

### Participants

Patients with AECOPD who might meet the inclusion criteria will be screened in the outpatient and inpatient wards, and informed consent will be signed for all patients enrolled in the study. The inclusion criteria are as follows:

- A confirmed diagnosis of moderate to very severe AECOPD
- Age between 40 and 80 years
- Syndrome differentiation meeting criteria of syndrome of external cold and internal fluid, syndrome of phlegm-heat congesting the lung, or syndrome of phlegm-damp amassing in the lung
- No participation in other interventional trials in the previous one month
- Provision of signed informed consent

The exclusion criteria are as follows:

- Pregnant and lactating women
- Dementia, mental disorders, and reluctant partners
- Complicated with heart failure (New York Heart Association [NYHA] Class IV) serious cardiac arrhythmias, or unstable hemodynamics
- Current respiratory disorders other than COPD (e.g., bronchiectasis, active tuberculosis, pneumothorax, pleural effusion, pulmonary thromboembolism, or neuromuscular diseases affecting respiratory movement function)
- Combined tumor
- Treatment outside the hospital for more than 7 days
- Need to carry out invasive mechanical ventilation for respiratory failure
- Complicated with serious hepatic or renal diseases (liver cirrhosis, portal hypertension, bleeding of varicose veins, dialysis, or renal transplantation)
- Bedridden for various reasons
- Allergic to the used medicine

The rejecting criteria are as follows:

- Impacts assessment of effectiveness and safety of the study by seriously violating the research plan
- There is no way to judge efficacy and safety due to lack of data

### Withdrawal, dropout, and discontinuation

Participants can refuse to participate in the study and follow-up evaluation after enrollment. Other indications for withdrawal are the following situations: (1) development of serious disease (such as acute respiratory failure, acute myocardial infarction, acute heart failure, or stroke) during the observation period; (2) receiving other CM for AECOPD during the observation period; (3) participants who present with serious adverse events (such as death, disability, surgery, or congenital malformations) (4) participants who refuse to continue to participate in the RCT; (5) those lost to follow-up.

### Intervention

#### Health education

The aim of health education is to improve the patient and family awareness of COPD, improve their ability to cope with COPD, and better coordinate treatment and prevention. The substance includes (1) education and supervision and urging patients to stop smoking, guidance to quit smoking, providing means to quit smoking, and striving to reduce passive smoking; (2) mastering the general and certain specific treatment methods, urging patients to adhere to treatment; (3) learning to control CPOD, e.g., with abdominal breathing and shrinkage lip breathing; (4) understanding the timing of hospital visits; (5) avoiding or preventing inhalation of dust, smoke, and harmful gases, etc.

#### Conventional drug

All subjects recruited will receive conventional drug treatment based on the 2017 Global Initiative for Chronic Obstructive Lung Disease (GOLD).

#### Chinese medicine

On the basis of health education and conventional drug treatment, the intervention group will be treated with CM, and the control group will be given a CM placebo. According to traditional CM syndromes, Sanhanhuayin granules are given for the syndrome of external cold and internal fluid, Qingrehuatan granules for the syndrome of phlegm-heat congesting the lung, and Zaoshihuatan granules for the syndrome of phlegm-damp amassing in the lung. Both the CM and the CM placebo granules come in bags, 10 g/bag, which are produced by Jiangyin Tian Jiang Pharmaceutical Co., Ltd. Test results of drug quality were consistent with the required quality standards. Each type of granule will be given orally, twice a day, for 14 days. The dose of the drug is not allowed to be modified.

### Combination therapy

During the treatment period, if there is comorbidity, certain symptomatic treatments can be given according to specific clinical conditions, but it is forbidden to use traditional CM or Chinese patent medicines that provide results similar to the research effect. Drugs for coronary heart disease, diabetes, and hypertension are all prescribed according to the guidelines for the disease. The name, manufacturer, batch number, usage, dosage, etc., of the drug will be recorded in detail.

### Outcomes

#### Primary outcome: COPD Assessment Test (CAT)

The primary outcome of this study will be changes in the CAT between baseline, 14 days after treatment, and the 28 and 42 days follow-ups. We will use the CAT to evaluate the impact of AECOPD on a person’s life and how this changes over time.

The CAT is an 8-item questionnaire designed to assess and quantify health-related quality of life and symptom burden in patients with COPD [10, 11]. Each questionnaire item is presented as a semantic 6-point (0–5) differential scale, providing a total score out of 40. Scores of 0–10, 11–20, 21–30, 31–40 represent mild, moderate, severe, or very severe clinical impact, respectively [12]. Evidence has shown the CAT may also prove useful in clinical trials to objectively assess the ability of novel interventions to reduce AECOPD severity [13], and it is a potentially useful instrument to assess the efficacy of treatments following COPD exacerbations [14].

#### Secondary outcomes

The secondary outcomes are described as follows:

1. Treatment failure rate: Treatment failure is defined as a worsening of symptoms and signs or death [15]. The outcome is the treatment failure rate on day 14 after admission.
2. Treatment success rate: Treatment success is defined as cure (a complete resolution of signs and symptoms associated with the exacerbation) or improvement (a resolution or reduction of the symptoms and signs associated with the exacerbation, without new symptoms or signs) [15]. The outcome is the treatment success rate on day 14 after admission.
3. Length of hospital stay: The length of hospital stay will be recorded at 14 days of the treatment phase.
4. Readmission due to AECOPD: The number of readmissions due to AECOPD will be recorded in the 28-day follow-up period.
5. Intubation rate: The number of intubations will be recorded during hospitalization and in the 28-day follow-up period.
6. Mortality: The number of deaths will be recorded during hospitalization and in the 28-day follow-up period.
7. Dyspnea: The Modified British Medical Research Council (mMRC) questionnaire [16] was considered adequate for assessment of dyspnea, as the mMRC relates well to other measures of health status [17] and predicts future mortality risk [18]. It will be completed and recorded at days 0, 4, 7, 10, and 14 of the treatment phase and at days 28 and 42 of the follow-up phase.
8. Quality of life: The Chinese version of the 36-item Short Form Health Survey (SF-36) [19] and COPD patient-reported outcome scale (COPD-PRO) [20] will be adopted to assess the health-related quality of life of patients with AECOPD.
  The SF-36 contains eight domains: physical function, restrictions in activity due to physical problems, bodily pain, general health, vitality, social function, restrictions in activity due to emotional problems, and mental health. The COPD-PRO has inherent correlation with the evaluation of efficacy of CM based on clinical symptoms. It contains 17 items in three domains: amelioration of clinical symptoms, satisfaction of health condition, and satisfaction of treatment effect [20].

Each participant will be assessed by the investigator at baseline, 14 days after treatment, and at the 28 days and 42 days follow-ups.

### Safety assessment

Participants will be asked to report any adverse events or related information during the treatment phase and the follow-up phase. The details of every adverse event will be reported in the Case Report Form.

### Data management

The Independent Data Monitoring Committee (IDMC) is a group of clinicians and biostatisticians appointed by study sponsors who provide independent assessment of the safety, scientific validity, and integrity of clinical trials [21]. The IDMC members of this study include two clinicians and one biostatistician with expertise in clinical trial and interim data analysis. The functions and frequency of its meetings are dictated by the DMC charter. The IDMC can recommend premature closure or reporting of the trial.

In order to improve patient compliance, we have taken the following measures: (1) we explain the purpose, significance, and related follow-up time of the study before enrollment; (2) we perform regular telephone follow-up to be informed of the changes in the subject’s condition and provide necessary assistance.

Jiangsu Famous Medical Technology Co., Ltd. in Nanjing, China is responsible for managing the data (e.g., plans for data entry, coding, security, and storage, double data entry; range checks for data values), formulating statistical analysis plans, writing statistical analysis reports, and final review by the project responsible unit.

### Statistical analysis

All data will be analyzed according to the intention-to-treat (ITT) and per-protocol (PP) analyses to reduce deviation. The modified intention-to-treat (mITT) analysis set is a subset of the ITT population and allows the exclusion of some randomized subjects in a justified way. The PP analysis set will include patients who fully complete the trial. The mITT analysis set is used for efficacy and the PP analysis set for safety. Continuous variables will be presented as mean and standard deviation or median and interquartile range according to the Kolmogorov-Smirnov test, and categorical variables as number of patients and proportion.

The Student’s *t* test will be used to compare differences in measurement data. The rank sum test will be used to deal with ranked data, the chi-squared test will be used to analyze categorical data, and the repeated measures analysis of variance (ANOVA) test will be used to analyze multiple measurements at different time-point data. If the effect of the different centers is significant, the Cochran-Mantel-Haenszel (CMH) chi-square test or analysis of covariance (ANCOVA) test is used to correct it. A statistician not involved in data collection will conduct all statistical analyses using SPSS version 23.0 software (SPSS Inc., Chicago, IL, USA).

### Clinical trial registration

This trial was registered at ClinicalTrials.gov on 4 February 2018 (NCT03428412).

## Discussion

AECOPD are important events in the management of COPD because they negatively impact health status and rates of hospitalization and readmission, and potentially result in death and functional disability [22, 23]. The goals of treatment for AECOPD are to minimize the negative impact of the current exacerbation and prevent the development of subsequent events. The chemical medications most commonly used for AECOPD are bronchodilators, corticosteroids, and antibiotics. CM is used extensively in the treatment of AECOPD in Asia, particularly in China. The effects of CM, especially herbal medicines, in improving respiratory symptoms and decreasing the incidence of COPD exacerbations are well known.

Clinical studies have postulated that herbal medicine is effective as a supplemental remedy for treating AECOPD [8, 24]. However, due to the single type of syndrome, limited sample sizes, or methodological problems apparent in some studies, the evidence for the effectiveness of CM was not robust. This RCT will investigate the effectiveness of CM in the treatment of AECOPD. We are attempting to conduct a clinical RCT with adequate design and few deficits. We performed a multicenter, randomized, double-blind, placebo-controlled clinical trial to assess the beneficial or harmful effects of CM for treating AECOPD.

An exacerbation of COPD is defined as an acute worsening of respiratory symptoms that result in additional therapy. The key symptom of an exacerbation is increased dyspnea. Other symptoms include increased sputum purulence and volume, together with increased coughing and wheezing [25]. We thought that if CM is effective in reducing increased dyspnea, cough, and sputum caused by exacerbation, it may primarily affect the respiratory symptoms, so we consider this as the primary outcome of our trial. We also examine the incidence of treatment failure and intubation, length of hospital stays, readmission due to exacerbation, number of deaths, and quality of life to obtain more evidence for AECOPD with CM. Therefore, we hope this trial will provide useful evidence for developing a therapeutic schedule with CM for patients with AECOPD.

### Trial status

The current protocol is version 8.0, 16 October 2018. The study began recruiting in August 2018. At the time of manuscript submission, we have recruited 36 patients. The recruitment of participants has now been completed. Follow-up assessments of patients are expected to be completed in January 2020. A populated SPIRIT checklist is available as Additional file 1.

## Supplementary information

**Additional file 1.** SPIRIT 2013 checklist: recommended items to address in a clinical trial protocol and related documents.

## Abbreviations

AECOPD: Acute exacerbations of COPD
CAT: COPD Assessment Test
CM: Chinese medicine
COPD: Chronic obstructive pulmonary disease
COPD-PRO: COPD patient-reported outcome scale
GOLD: Global Initiative for Chronic Obstructive Lung Disease
mMRC: Modified (British) Medical Research Council
RCT: Randomized controlled trial
SF-36: 36-item Short Form Health Survey
